# Gene family–level endocrine compensation and metabolic reprogramming preserve growth homeostasis under sustained insulin-like peptide suppression in *Litopenaeus vannamei*

**DOI:** 10.3389/fendo.2026.1842807

**Published:** 2026-06-08

**Authors:** Jiawei Liu, Zijie Liu, Xiaowei Song, Su Liu, Lin Song, Yi Gao

**Affiliations:** 1 School of Marine Science and Engineering, Qingdao Agricultural University, Qingdao, China; 2 Shandong Provincial Key Laboratory of Biochemical Engineering, College of Biological Engineering, Qingdao University of Science and Technology, Qingdao, China; 3 Wuqiong Food Co., Ltd., Raoping, China

**Keywords:** endocrine compensation, glucose homeostasis, insulin-like peptides (ILPs), *Litopenaeus vannamei*, metabolic reprogramming

## Abstract

Insulin-like peptide (ILP) signaling is a critical endocrine axis governing growth, metabolism, and reproduction in crustaceans. In decapods, the ILP family has diversified into four subtypes through gene duplication, yet the functional coordination among these ligands to buffer endocrine perturbations remains poorly understood. Building on our previous identification of insulin-type ILP (*LvIns*) as a key regulator of growth and glucose metabolism in *Litopenaeus vannamei*, we investigated the long-term physiological consequences of sustained *LvIns* suppression. Surprisingly, prolonged knockdown did not result in progressive growth deterioration or elevated mortality. Instead, shrimp exhibited a notable recovery of growth and metabolic performance, revealing a dynamic adaptive response to chronic endocrine perturbation. Notably, this recovery coincided with robust up-regulation of the gonadulin-type ILP *LvGon*, another ILP family member. *In vivo* glucose challenge and exogenous insulin assays, together with *in vitro* primary hepatopancreas cell experiments, demonstrated that *LvGon* is rapidly induced by hyperglycemia and suppressed by insulin, placing it within a glucose–insulin regulatory loop. *LvGon* also exhibited broad tissue distribution similar to *LvIns*, sharing conserved insulin-like structural features and predicted insulin receptor–binding potential. Transcriptomic analysis further revealed coordinated metabolic reprogramming, including up-regulation of glycolysis, fatty acid oxidation/ketogenesis, and the tricarboxylic acid cycle, as well as activation of growth-associated Wnt and MAPK/ERK pathways. Collectively, these findings support a multilevel compensatory framework, in which ligand-level substitution (*LvGon* up-regulation), metabolic remodeling, and growth signaling activation converge to restore growth homeostasis under sustained disruption of insulin-like signaling, providing mechanistic insight into endocrine redundancy and network plasticity in crustaceans.

## 1 Introduction

The insulin/insulin-like growth factor signaling (IIS) pathway is a highly conserved regulatory axis in metazoans (1, 2), orchestrating fundamental physiological processes including metabolic homeostasis, growth, development, and reproduction (3, 4). It is initiated by the binding of peptide ligands to insulin receptors, thereby triggering conserved intracellular signaling cascades such as the PI3K–AKT pathway (5). In mammals, IIS is primarily activated by three types of ligands: insulin, insulin-like growth factors (IGFs) and relaxins (6). In contrast, invertebrate IIS is predominantly mediated by insulin-like peptides (ILPs), which serve as functional analogs of mammalian insulin (7). Notably, invertebrates exhibit remarkable expansion and diversification of ILPs, with gene copy numbers ranging from three in orthopteran locusts to 38 in lepidopteran silkworms, and up to 40 members in *Caenorhabditis elegans* (8–10). These findings suggest that the ILP family has undergone extensive expansion and functional diversification throughout invertebrate evolution (11).

Gene family expansion provides the molecular basis for functional diversification (12), but it also raises a fundamental biological question: when the function of a core ligand is impaired, can paralogous members maintain pathway stability and physiological integrity through compensatory regulation and functional redundancy? Such compensatory plasticity is particular relevance to insulin signaling (13). In mammals, the insulin-encoding gene exists as a single copy; consequently, insulin resistance induced by hyperglycemia or metabolic stress is primarily counteracted through pancreatic β-cell proliferation and increased insulin secretion, representing a compensation model centered on a single ligand (14, 15). In contrast, invertebrates harboring multiple ILP paralogs may employ more flexible and distributed compensatory strategies (7). Accumulating evidence indicates that loss of specific ILPs can be buffered by transcriptional up-regulation of other family members. For instance, the loss of *DILP2*, *DILP3*, and *DILP5* in the *Drosophila* brain induces the up-regulation of *DILP6* in the fat body, partially restoring systemic insulin signaling (3). Knockdown of *DILP2* alone similarly triggers compensatory increases in *DILP3* and *DILP5* transcripts (16). Comparable patterns have been reported in other insects, including *Callosobruchus maculatus*, in which silencing *CmILP1* or *CmILP2* results in the up-regulation of *CmILP3* expression (17), and in *Blattella germanica*, knockdown of *BgILP5* triggers an increase in *BgILP3* levels within the brain (18). Collectively, these findings highlight a compensation model based on functional complementation among ILP family members, which enhances both pathway robustness and evolutionary adaptability.

Crustaceans, the sister clade to insects, represent a major invertebrate lineage with critical ecological roles and substantial economic importance in global aquaculture (19). Similar to insects, crustaceans possess multiple copies of the ILP gene family (20). To date, four distinct ILPs types have been identified in decapod crustaceans: insulin-type, relaxin-type, gonadulin (Gon)-type, and insulin-like androgenic gland factor (IAG)-type (21). Previous studies have demonstrated that ILP signaling broadly regulates growth, metabolism, and reproduction in crustaceans (22, 23). Among them, IAG has been established as a pivotal regulator of male sexual differentiation and gonadal development (24). Gon-type ILP was first discovered in the gonads of *Penaeus japonicus*, with subsequent studies showing its involvement in glucose regulation in *Portunus trituberculatus* and *Macrobrachium rosenbergii* (25–27). Relaxin-type ILP has been shown to be involved in the regulation of shrimp growth, molting, and glucose metabolism (22, 28). Insulin-type ILPs were initially identified in the ovarian transcriptome of *Marsupenaeus japonicus* and have been shown to play a role in ovarian development (26). Despite these advances, most studies have examined individual ILP types in isolation, and systematic analyses of functional interactions and compensatory relationships among ILP family members remain scarce (29, 30).

In our previous work, we identified an insulin-type ILP (*LvIns*) in *Litopenaeus vannamei*, which is homologous to *Drosophila DILP1*–*6* (31). Functional and metabolic interference experiments demonstrated that *LvIns* is a critical regulator of shrimp growth and glucose homeostasis, with *LvIns* knockdown leading to a marked reduction in growth rate within 12 days (31). Building upon these findings, we extended the duration of *LvIns* interference to further examine its long-term physiological effects. Unexpectedly, prolonged interference for 20 days did not result in sustained growth suppression; instead, shrimp exhibited partial weight recovery and accelerated growth, in contrast to the growth suppression observed during the first 12 days. Based on these findings, we hypothesize that prolonged suppression of *LvIns* activates an endogenous compensatory mechanism, thereby alleviating growth restriction and restoring physiological homeostasis.

To test this hypothesis, the present study extended the *LvIns* interference period and systematically examined its effects on the expression profiles of other ILP types, aiming to elucidate the endocrine regulatory mechanisms underlying ILP-mediated compensation. Pacific white shrimp (*L. vannamei*) is one of the most economically valuable aquaculture species worldwide, accounting for over 50% of the global shrimp production (32). Elucidating the compensatory interactions among diverse ILPs will advance our understanding of endogenous growth regulation in crustaceans. Additionally, this will provide broader insights into the evolutionary plasticity of endocrine networks in invertebrates.

## 2 Materials and methods

### 2.1 Experimental animals

Pacific white shrimp (*L. vannamei*) used in this study were obtained from a commercial aquaculture farm in Haiyang, Yantai, Shandong Province, China. Shrimp were acclimated for at least two weeks in a recirculating aquaculture system at the College of Marine Science and Engineering, Qingdao Agricultural University. During acclimation, water temperature was maintained at 23 ± 1 °C, which is within the species’ optimal thermal range. Continuous aeration was provided to ensure adequate dissolved oxygen. Shrimp were fed a commercial formulated shrimp diet (Tongwei Co., Ltd., China) three times daily at 08:00, 15:00, and 22:00. Uneaten feed and feces were removed each morning, and seawater was completely renewed daily. After acclimation, healthy shrimp were randomly selected and assigned to experimental groups to minimize potential variation due to sex and developmental stage.

All experimental procedures involving animals were conducted in accordance with the guidelines of the Animal Care and Use Committee of Qingdao Agricultural University (Approval No. 2018–192).

### 2.2 RNA interference of *LvIns*

Double-stranded RNA (dsRNA) targeting *LvIns* and enhanced green fluorescent protein (EGFP, used as a negative control) was synthesized following the method described by Guo et al. (2019) (33). Briefly, a fragment of the *LvIns* gene was amplified by PCR using gene-specific primers containing the T7 promoter sequence (**Table 1**) to generate the transcription template. *In vitro* transcription was performed using the TranscriptAid™ T7 High Yield Transcription Kit (Thermo Fisher Scientific, USA). The resulting dsRNA was purified by phenol–chloroform extraction, and its concentration and purity were determined using a NanoDrop spectrophotometer.

**Table 1:** Primer sequences and GenBank accession numbers of target genes used in this study.

Primer name	Primer sequence (5′-3′)	Accession no.
RNAi primers		
dsLvIns-F	TAATACGACTCACTATAGGGTCGCCCTGGTGTGTCTCC	PV469411.1
dsLvIns-R	TAATACGACTCACTATAGGGCGGAGTGCTGTAGGAAGGC	
dsEGFP-F	TAATACGACTCACTATAGGGCAGTGCTTCAGCCGCTACCC	
dsEGFP-R	TAATACGACTCACTATAGGGAGTTCACCTTGATGCCGTTCTT	
Quantitative primers		
QLvIns-F	TTCCTCGCCCTGGTGTGT	PV469411.1
QLvIns-R	CAAACTGAACAGCGTCTGCAA	
QLvGon-F	GGCGTTCTGTGCGTTCCT	PX548030.1
QLvGon-R	CCTCCTTGTCCCGCTTGAT	
QLvIAG-F	CTCGGATTGCTGATGCTTCTC	KM066114.1
QLvIAG-R	ATCGGCTGACCTTGACACTCT	
QLvRelaxin-F	CGGCGGAGTCTGATGAGGT	ON973859
QLvRelaxin-R	CAGCACTCGGTGGTGATGG	
QLv18S-F	TATACGCTAGTGGAGCTGGAA	AF186250.1
QLv18S-R	GGGGAGGTAGTGACGAAAAAT	
QLvG6PI-F	GAGTTCTGGGACTGGGTT	LOC113819923
QLvG6PI-R	CAAGCAATACAGGAATGTTC	
QLvPK-F	GAAGCCTCATTCAAAGCA	LOC113828822
QLvPK-R	CAGCATTCTGAGGCACAG	
QLvPDHB-F	GTGATGCCCTCAACTCCG	LOC113817644
QLvPDHB-R	GCTCCAACAGCAATACCG	
QLvGAPDH-F	GTTCAAGTATGACAGCACCC	LOC113828374
QLvGAPDH-R	CAGTGGACTCAACGATGTATT	
QLvGLUT1-F	ATTGTGACCCTCTATGTGG	LOC113810126
QLvGLUT1-R	ACTGACCCTGTACCCTTG	
QLvGBE-F	AGGTTCCGAAGATCAACGAC	LOC113821820
QLvGBE-R	ATGCCAAAGGTCTCATAGCC	
QLvHSC70-4-F	CCAAGTTAGACAAGAGTAGTATCC	LOC113805260
QLvHSC70-4-R	TGTTGCGTTTGATGAGGG	
QLvPEPCK-F	TCATCTCCTTCGGGTCGG	NewGene_933
QLvPEPCK-R	GCCCACGCACTCCACTTT	
QLvNFIL3-F	TTGCCTCGTCATATCTCACC	LOC113813388
QLvNFIL3-R	AGGACTTCTTTGGGAATTACTCT	

Forward and reverse primers are denoted by F and R, respectively.

Based on preliminary dose optimization experiments, 4 μg dsLvIns (dissolved in 10 μL sterile PBS) was selected as the effective interference dose. Healthy shrimp of uniform size were used for 12-day and 20-day RNA interference (RNAi) experiments.

For the 12-day experiment, shrimp with an initial average body weight of 2.14 ± 0.22 g (n = 180) were randomly divided into three groups: PBS control group (10 μL PBS), dsEGFP negative control group (4 μg/10 μL), and dsLvIns interference group (4 μg/10 μL). Each group consisted of three biological replicates with 20 shrimp per replicate. All injections were administered into the abdominal intersegmental muscle. To maintain sustained gene silencing, injections were repeated every four days, based on the RNAi interference efficiency and our preliminary experiments. Body weight was measured prior to each injection (i.e., every four days) to monitor growth dynamics.

The 20-day interference experiment was conducted as an extension of previous work (31). Shrimp with an initial average body weight of 1.19 ± 0.24 g were injected with dsLvIns, dsEGFP, or PBS every four days. Body weight was recorded from day 12 onward using the same procedure as described above. Growth rate was calculated using body weight on day 1 (for the 12-day experiment) or day 12 (for the 20-day experiment) as the baseline. Growth rate was expressed as the percentage increase relative to the corresponding baseline weight. At the end of the experiment, 24 shrimp were randomly selected from both the PBS control group and the dsLvIns interference group and divided into three biological replicates. Hepatopancreas tissues were collected under sterile conditions, immediately frozen in liquid nitrogen, and stored at −80 °C for subsequent analyses.

### 2.3 Determination of hepatopancreatic glucose content and glucose metabolism enzyme activities

Hepatopancreatic glucose content was measured using samples collected at 2 days (from the 12-day experiment), 12 days, and 20 days post-interference as described in Section 2.2. All assays were performed using commercial kits from Nanjing Jiancheng Bioengineering Institute (China) according to the manufacturer’s instructions. Glucose concentration in hepatopancreas homogenate supernatants from the PBS control and dsLvIns groups was determined using a glucose assay kit (Cat. No. F006-1-1; Nanjing Jiancheng Bioengineering Institute, China).

Enzyme activity assays were conducted using hepatopancreas samples collected after 20 days of interference. Activities of hexokinase (HK; Cat. No. A077-3-1; Nanjing Jiancheng Bioengineering Institute, China), phosphofructokinase (PFK; Cat. No. A129-1-1; Nanjing Jiancheng Bioengineering Institute, China), and phosphoenolpyruvate carboxykinase (PEPCK; Cat. No. A131-1-1; Nanjing Jiancheng Bioengineering Institute, China) were measured following the manufacturer’s protocols.

### 2.4 RNA extraction, cDNA synthesis, and quantitative real-time PCR analysis after *LvIns* interference

Hepatopancreas samples collected after 20 days of *LvIns* interference (Section 2.2) were ground into powder under liquid nitrogen. Total RNA was extracted using RNAiso Plus reagent (TaKaRa, Japan) according to the manufacturer’s instructions. RNA concentration and purity were determined using a NanoDrop spectrophotometer (Thermo Fisher Scientific, USA), and RNA integrity was verified by 1% agarose gel electrophoresis. First-strand cDNA was synthesized from total RNA using the PrimeScript™ RT Reagent Kit (TaKaRa, Japan) following the manufacturer’s protocol. The synthesized cDNA was stored at −20 °C until further use.

Quantitative real-time PCR (qRT-PCR) was performed to determine the relative expression levels of four ILPs in *L. vannamei*, including *LvIns*, *LvGon*, *LvIAG*, and *LvRelaxin*. Gene-specific primers were designed based on previously obtained sequencing fragments of the four ILP genes. Amplification reactions were carried out using a QuantStudio™ 5 Real-Time PCR System (Thermo Fisher Scientific, USA) with TB Green^®^ Premix Ex Taq™ II (TaKaRa, Japan) as the fluorescent dye. The thermal cycling conditions were as follows: initial denaturation at 95 °C for 3 min, followed by 40 cycles of 95 °C for 10 s, 55 °C for 30 s, and 72 °C for 30 s. The 18S rRNA gene was used as the internal reference for normalization. Primer sequences and GenBank accession numbers are listed in **Table 1**. Each sample was analyzed in triplicate (technical replicates), and relative gene expression levels were calculated using the 2^-ΔΔ^Ct method (34).

### 2.5 Semi-quantitative PCR analysis of tissue expression of ILPs

To determine the tissue distribution patterns of four ILPs in *L. vannamei*, 12 tissues were collected for semi-quantitative PCR analysis, including ovary (Ova), ventral nerve (Ven), intestine (Gut), stomach (Sto), eyestalk (Eye), testis (Tes), epidermis (Epi), hepatopancreas (Hep), heart (Hea), gill (Gil), hemocytes (Hem), and muscle (Mus). Total RNA extraction and cDNA synthesis for each tissue were performed as described in Section 2.4. PCR amplification of 18S rRNA was first conducted as an internal reference. PCR products were separated by 1% agarose gel electrophoresis, and band intensities were quantified to normalize cDNA template concentrations across samples. Equalized cDNA templates were subsequently used for gene-specific PCR amplification of the four ILPs. The primer sets were identical to those used for qRT-PCR (**Table 1**). After electrophoresis, the band intensities of target genes were quantified using ImageJ software (NIH, USA) based on integrated density values after background subtraction.

The expression level of *LvIns* in ovary was set as 100% and used as the reference for normalization. Relative band intensities of the other ILPs in different tissues were normalized accordingly. The resulting data were used to generate a tissue expression profile illustrating the distribution patterns of the four ILPs across the 12 examined tissues.

### 2.6 *In vivo* glucose injection assay and analysis of *LvGon* expression

The *in vivo* glucose injection experiment was conducted following the method described by Liu et al. (2026) (31). Briefly, shrimp with an average body weight of 20.45 ± 2.58 g were selected and fasted for 12 h prior to treatment to standardize metabolic status. The glucose treatment group was injected intramuscularly with glucose solution at a dose of 1.0 mg/g body weight, while the control group received an equal volume of PBS. Hemolymph samples were collected at 5, 15, 30, 60, 180, and 360 min post-injection. At each time point, hemolymph was collected from 36 shrimp, with samples from 12 individuals pooled to form one biological replicate. Hemolymph samples were centrifuged at 4 °C and 1000 rpm for 5 min to obtain serum. Glucose concentration was measured using a glucose assay kit (Cat. No. F006-1-1, Nanjing Jiancheng, China). Hemocyte pellets, hepatopancreas, stomach, and muscle tissues were also collected. Total RNA was extracted from tissues and hemocytes as described in Section 2.4, followed by cDNA synthesis. The dynamic expression of *LvGon* was analyzed by qRT-PCR.

### 2.7 *In vivo* exogenous insulin injection assay and analysis of *LvGon* expression

The *in vivo* insulin injection experiment was performed according to Liu et al. (2026) (31). A total of 360 shrimp with an average body weight of 10.93 ± 2.08 g were randomly divided into two groups and fasted for 12 h prior to treatment. Both groups were first injected intramuscularly with glucose solution (1.0 mg/g body weight). Ten minutes later, the experimental group received an additional injection of bovine insulin (8.0 IU/kg; G-Clone, Beijing, China), while the control group received an equal volume of PBS. Bovine insulin was employed as an exogenous insulin stimulus, based on previous studies in crustaceans demonstrating its biological activity in regulating hemolymph glucose levels (28, 35, 36). Samples were collected at 15, 30, 60, 180, and 360 min post-injection. At each time point, 36 shrimp were sampled per group, and hemolymph from 12 individuals was pooled to generate one biological replicate. Hemolymph, hepatopancreas, stomach, and muscle tissues were collected. Serum glucose levels were measured as described in Section 2.6. RNA extraction, reverse transcription, and qRT-PCR were performed following the procedures described in Section 2.4 to assess changes in *LvGon* expression.

### 2.8 Primary hepatopancreas cell culture, treatment, and *LvGon* expression analysis

#### 2.8.1 Cell isolation and culture

Following the protocols described by Liu et al. (2026) and Liu et al. (2024) (31, 36), hepatopancreas tissues were aseptically collected from 12 healthy shrimp. After mechanical dissociation, the tissue was passed through a 100 μm cell strainer to obtain a single-cell suspension. The cells were washed and resuspended in Leibovitz’s L-15 medium (Biosharp, China) supplemented with glutamine, and seeded into a 24-well cell culture plate (950 μL per well). Cells were cultured at 25 °C for 24 hours to stabilize their condition.

#### 2.8.2 Glucose treatment experiment

After stabilizing cell culture conditions, 50 μL of glucose solution was added to each well of the experimental group to achieve a final glucose concentration of 10 mg/mL, while the control group received an equal volume of PBS. Cells were collected by centrifugation at 1000 rpm for 5 minutes at 10, 20, and 40 minutes post-treatment. A portion of the cells was used for glucose content determination, following the protocol described in Section 2.3. The remaining cells were processed for RNA extraction and cDNA synthesis as described in Section 2.4, and the expression levels of *LvGon* were analyzed by qRT-PCR.

#### 2.8.3 Insulin treatment experiment

Cells in each well were initially treated with 50 μL of glucose solution (final concentration 10 mg/mL) for 10 minutes. Following this, the experimental group received 50 μL of bovine insulin solution (1.6 IU/mL), while the control group was treated with an equal volume of PBS. Cells were collected by centrifugation at 1000 rpm for 5 minutes at 10, 20, and 40 minutes post-treatment. A portion of the cells was used for glucose content determination (as described in Section 2.3). The remaining cells were processed for RNA extraction, cDNA synthesis, and subsequent *LvGon* expression analysis by qRT-PCR, as detailed in Section 2.4.

### 2.9 Sequence feature analysis of *LvIns* and *LvGon* and structural prediction of their interaction with the insulin receptor

Based on the obtained *LvIns* and *LvGon* sequences and their derived amino acid sequences, functional domain analysis was performed using the SMART (http://smart.embl.de/) and Pfam (https://pfam.xfam.org/) databases. Multiple sequence alignment was conducted using MEGA software (Version 11, New Zealand) to identify conserved cleavage sites in the ILP precursors and conserved cysteine residues.

Further structural predictions of the interaction between *LvIns* and *LvGon* with the insulin receptor (NCBI accession number: PP932464.1) were performed using AlphaFold3 (https://alphafoldserver.com/about?golgi=true). The predicted complexe showed ipTM value above 0.8, supporting high confidence in the predicted interaction models. The three-dimensional structures and interaction patterns were visualized using PyMOL (Version 2.4, USA) (37, 38).

### 2.10 Transcriptome sequencing and bioinformatic analysis following *LvIns* interference

RNA sequencing (RNA-Seq) was performed on hepatopancreas tissues collected after 20 days of *LvIns* interference as described in Section 2.2. The detailed procedures were as follows.

Nine hepatopancreas samples were randomly selected from both the dsLvIns interference group and the PBS control group. Every three samples were pooled to generate one biological replicate, resulting in three biological replicates per group and a total of six sequencing libraries. Total RNA was extracted using the RNAprep Pure Plant Kit (Tiangen, China). RNA concentration and purity were assessed using a NanoDrop 2000 spectrophotometer (Thermo Fisher Scientific, USA), and RNA integrity was evaluated using an Agilent 2100 Bioanalyzer (Agilent Technologies, USA). For qualified RNA samples, mRNA was fragmented and reverse-transcribed to synthesize first-strand cDNA, followed by end repair and adaptor ligation to construct sequencing libraries. Paired-end sequencing (PE150) was performed on the Illumina NovaSeq 6000 platform (Biomarker Technologies, China).

Raw sequencing reads were processed to remove adaptor sequences, poly-N reads, and low-quality reads to obtain high-quality clean reads. Clean reads were aligned to the *L. vannamei* reference genome (version: GCF_003789085.1) using HISAT2. Gene expression levels were normalized and quantified as FPKM (Fragments Per Kilobase of transcript per Million mapped reads). Differentially expressed genes (DEGs) were identified using DESeq2 with thresholds of |Fold Change| ≥ 1.5 and false discovery rate (FDR) < 0.05. Gene Ontology (GO) enrichment analysis was conducted using the clusterProfiler package, and KEGG pathway enrichment analysis was performed using the KOBAS database in combination with clusterProfiler.

To validate the reliability of RNA-Seq results, nine representative DEGs were selected for qRT-PCR verification, including Glucose-6-phosphate isomerase (*G6PI*), Pyruvate kinase (*PK*), Pyruvate dehydrogenase E1 subunit beta (*PDHB*), Glyceraldehyde-3-phosphate dehydrogenase (*GAPDH*), Facilitated glucose transporter member 1 (*GLUT1*), 1,4-alpha-glucan branching enzyme (*GBE*), Heat shock 70 kDa protein cognate 4 (*HSC70-4*), Phosphoenolpyruvate carboxykinase (*PEPCK*), and Nuclear factor interleukin-3-regulated protein (*NFIL3*). Independent hepatopancreas samples from the dsLvIns and control groups were used for validation following the qRT-PCR procedure described in Section 2.4.

Relative gene expression levels were expressed as log_2_FoldChange (log_2_FC), and log_2_FC values obtained from qRT-PCR were compared with those derived from RNA-Seq analysis. The accession numbers and primer sequences of the selected genes are listed in **Table 1**.

### 2.11 Statistical analysis

All data are presented as mean ± standard deviation (SD). Statistical differences among groups were analyzed using one-way analysis of variance (ANOVA), followed by Duncan’s multiple range test for *post hoc* comparisons. Statistical significance was set at p < 0.05, and highly significant differences were defined as p < 0.01.

## 3 Results

### 3.1 *LvIns* knockdown significantly suppresses shrimp growth during early interference

**Figure 1:**
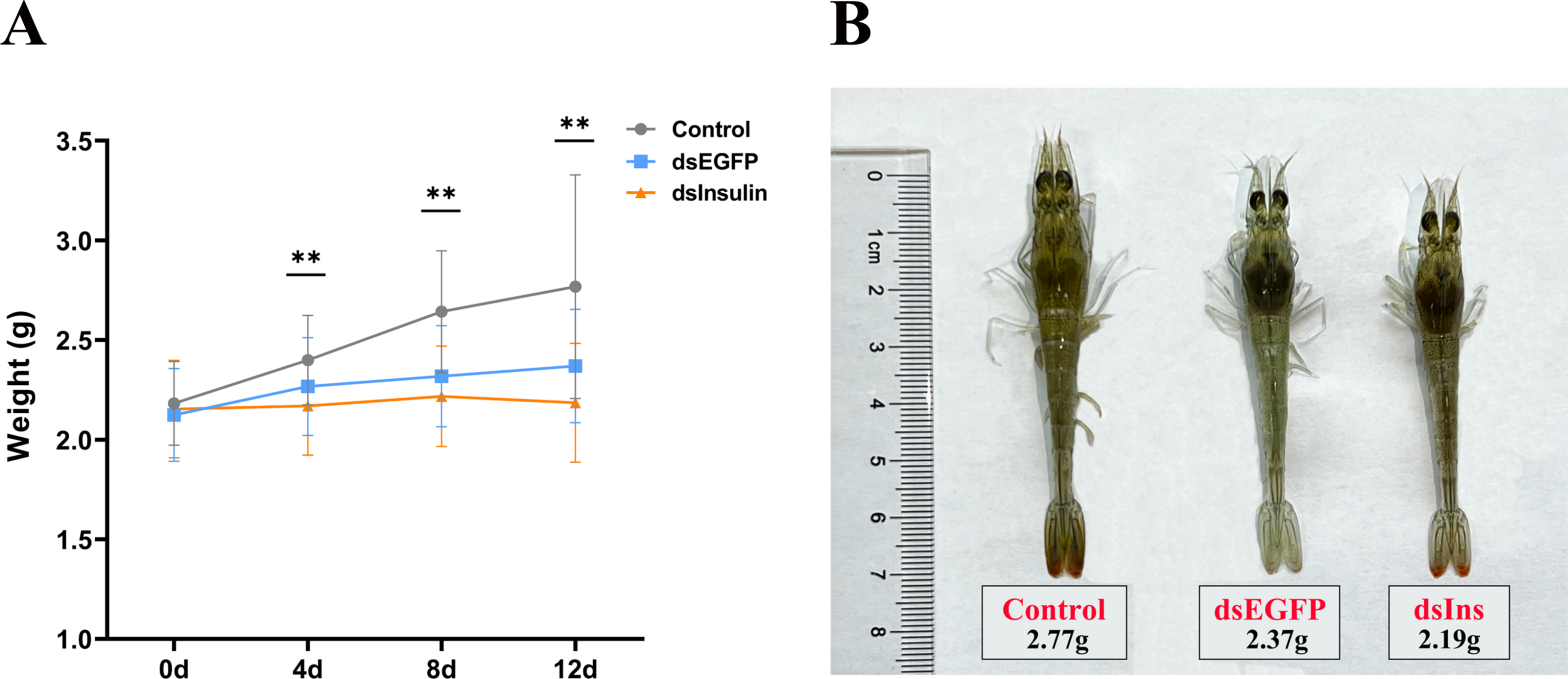
Effects of *LvIns* knockdown on growth performance in *L. vannamei* over a 12-day RNAi treatment. **(A)** Changes in mean body weight of shrimp subjected to continuous *LvIns* RNA interference during the experimental period. Data are presented as mean ± SD (n = 60 per group, with 3 biological replicates of 20 shrimp each). **p < 0.01 indicates significant differences between the dsLvIns group and both control groups at the corresponding time points. **(B)** Representative individuals from each treatment group at the end of the experiment (day 12). The selected shrimp correspond to individuals with body weights close to the group mean.

To validate the contribution of *LvIns* in growth of *L. vannamei*, we repeated our previously reported *LvIns* RNAi experiment (31). Shrimp were subjected to continuous *LvIns* RNAi for 12 days, with body weight measured every 4 days. Consistent with prior findings, *LvIns* knockdown significantly suppressed weight gain after 12 days (**Figure 1A**). After 12 days, the mean body weight of the *LvIns* RNAi group (2.19 ± 0.30 g) was significantly lower than that of both the PBS control (2.77 ± 0.56 g) and the dsEGFP negative control (2.37 ± 0.28 g). Consistent with these quantitative measurements, clear morphological differences were observed among treatment groups (**Figure 1B**). Notably, significant reductions in body weight were already detectable by day 4 of RNAi treatment, with the *LvIns* knockdown group averaging 2.17 ± 0.25 g compared with 2.40 ± 0.22 g in the PBS group and 2.27 ± 0.25 g in the dsEGFP group (p < 0.01). Collectively, these results demonstrate that *LvIns* is essential for normal growth in *L. vannamei*, and that its down-regulation leads to an early and sustained impairment of growth performance.

### 3.2 Effects of prolonged *LvIns* interference on shrimp growth

**Figure 2:**
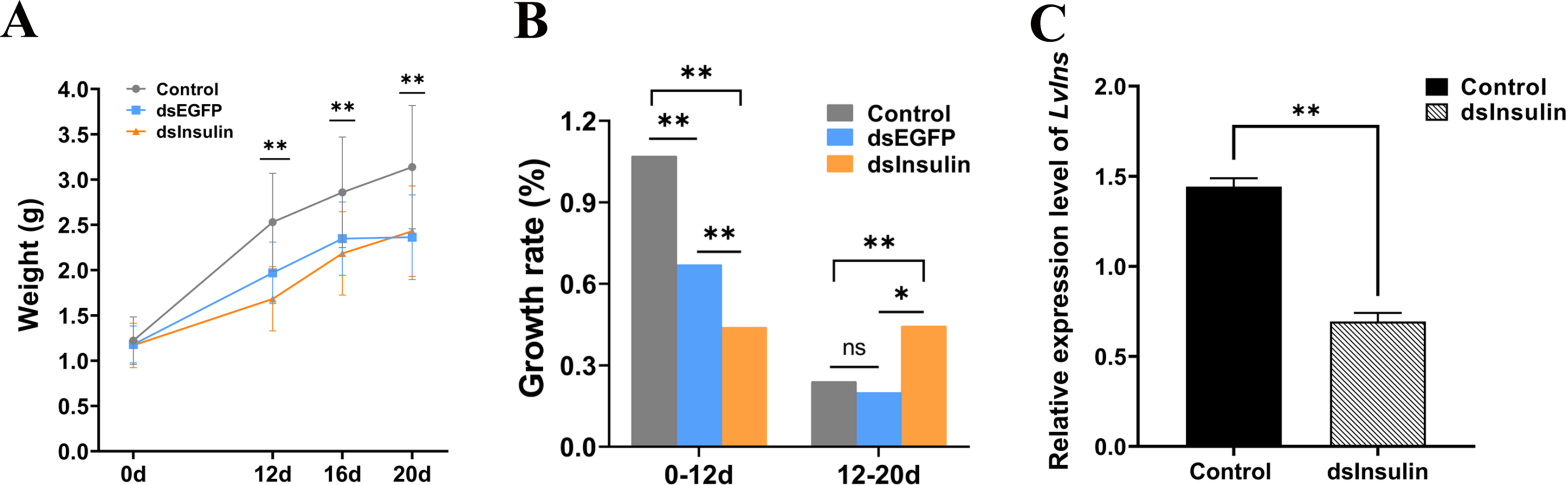
Effects of prolonged *LvIns* RNA interference on growth performance and gene expression in *L. vannamei.* **(A)** Temporal changes in mean body weight of shrimp subjected to continuous *LvIns* interference over a 20-day experimental period. Body weight was recorded at 0, 12, 16, and 20 days after the initiation of RNA interference (n = 60 per group, with 3 biological replicates of 20 shrimp each). **(B)** Comparison of growth rates during the early (0–12 days) and late (12–20 days) phases of the experiment among different treatment groups. **(C)** Relative expression level of the *LvIns* in the hepatopancreas after 20 days of RNA interference (n = 24 per group, with 3 biological replicates of 8 shrimp each). Data are presented as mean ± SD. Statistical significance is indicated as *p < 0.05, **p < 0.01, and ns denotes no significant difference.

Building on the initial 12-day RNAi experiment (31), we extended the *LvIns* interference period to 20 days, with mean body weight recorded at 4-day intervals (**Figure 2A**). During the first 12 days of interference, shrimp in the *LvIns* knockdown group exhibited significantly lower body weight than those in both the PBS control and dsEGFP negative control groups. Correspondingly, the growth rate of the *LvIns* interference group (44.00%) was markedly lower than that of the dsEGFP (67.00%) and PBS control groups (107.00%) (**Figure 2B**). Unexpectedly, from Day 12, shrimp in the *LvIns* interference group displayed a pronounced recovery in growth. Between Day 12 and Day 20, the growth rate in this group reached 44.44%, significantly exceeding that observed in the dsEGFP (19.95%) and PBS control groups (24.03%) (**Figure 2B**). By Day 20, the mean weight in the dsLvIns group (2.43 ± 0.50 g) even surpassed that of the dsEGFP group (2.36 ± 0.47 g). To confirm the persistence of the RNAi effect, qRT-PCR analysis was performed. The results showed that *LvIns* expression in the interference group remained significantly suppressed after 20 days, at only 48.08% of the control group’s level (**Figure 2C**), confirming that the interference effect was sustained throughout the experiment.

### 3.3 Effect of *LvIns* gene interference on glucose metabolism and the expression of other ILPs

**Figure 3:**
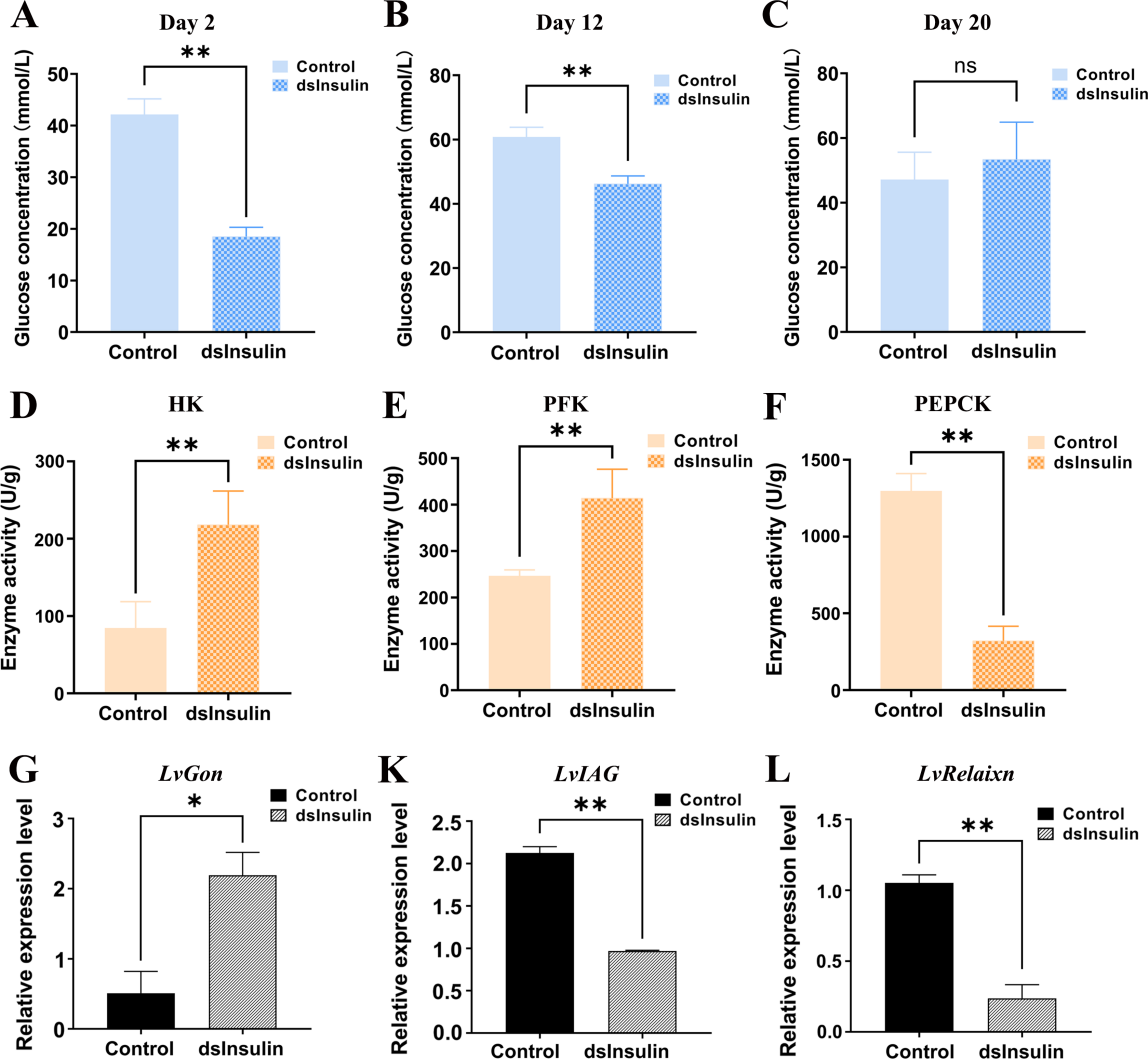
Effects of *LvIns* RNA interference on glucose metabolism related genes and enzymatic activities in the hepatopancreas of *L. vannamei*. **(A–C)** Changes in glucose concentration in the hepatopancreas at 2, 12, and 20 days following *LvIns* RNA interference, respectively. **(D–F)** Activities of key glucose metabolism related enzymes in the hepatopancreas after 20 days *LvIns* interference, including hexokinase (HK), phosphofructokinase (PFK), and phosphoenolpyruvate carboxykinase (PEPCK). **(G–L)** Relative expression levels of *LvGon*, *LvIAG*, and *LvRelaxin* in the hepatopancreas following 20 days of *LvIns* interference. Data are presented as mean ± SD (n = 24 per group, with 3 biological replicates of 8 shrimp each). Statistical significance is indicated as *p < 0.05 and **p < 0.01.

To further evaluate the metabolic consequences of *LvIns* interference, changes in hepatopancreatic glucose content were monitored at different time points. On Day 2 of interference, glucose levels in the hepatopancreas of the *LvIns* interference group were significantly reduced to 43.89% of those in the PBS control group (**Figure 3A**). By Day 12, hepatopancreatic glucose content partially recovered, reaching 75.97% of control levels (**Figure 3B**). Notably, after 20 days of interference, hepatopancreatic glucose levels in the *LvIns* knockdown group exceeded that of the control group, increasing to 113.22% (**Figure 3C**).

Key metabolic enzyme activities in the hepatopancreas were also significantly altered after 20 days of *LvIns* interference. The activities of the glycolysis rate-limiting enzymes hexokinase (HK) and phosphofructokinase (PFK) increased by 257.89% and 144.16%, respectively, compared with the control group (**Figures 3D,E**). In contrast, the activity of phosphoenolpyruvate carboxykinase (PEPCK), a key rate-limiting enzyme in gluconeogenesis, was markedly reduced by 75.21% (**Figure 3F**). Notably, this pattern of enzyme activity changes was opposite to the expression profiles of glucose metabolism–related genes previously observed at the end of the 12-day *LvIns* interference period (31).

In addition, the expression levels of other ILP genes in the hepatopancreas were assessed after 20 days of *LvIns* interference. *LvGon* expression was significantly up-regulated, reaching 4.30-fold that of the control group (**Figure 3G**), while the expression of *LvIAG* and *LvRelaxin* was significantly down-regulated (**Figures 3K, L**).

### 3.4 Effects of exogenous glucose administration on *LvGon* expression

**Figure 4:**
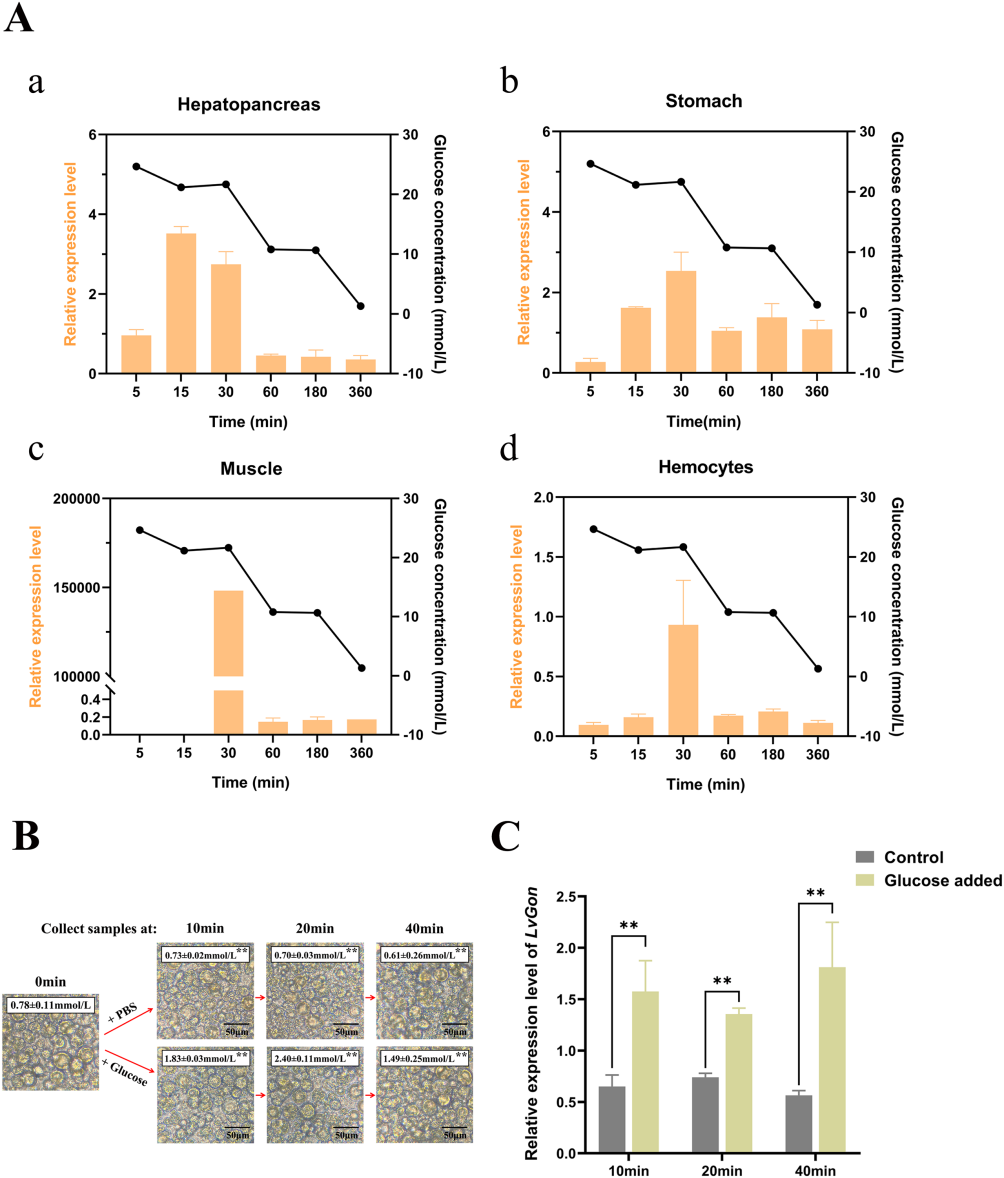
Effects of glucose administration on *LvGon* expression. **(A)** Changes in serum glucose concentration (black line) following *in vivo* glucose injection, together with the corresponding relative expression levels of *LvGon* (orange bars) in four tissues: (a) hepatopancreas, (b) stomach, (c) muscle, and (d) hemocytes. At each time point, data represent three biological replicates (12 shrimp per replicate). **(B)** Experimental design of the *in vitro* glucose treatment in primary hepatopancreatic cells and representative microscopic images. The values shown in the white boxes indicate the measured intracellular glucose concentrations. Asterisks (**) denote significant differences in glucose concentration between the glucose-treated group and the control group. **(C)** Relative expression levels of *LvGon* in primary hepatopancreatic cells after *in vitro* glucose treatment for different time points. For each treatment group at each time point, three independent replicate wells were established (n = 3), all derived from the mixed cell suspension of 12 shrimp. Data are presented as mean ± SD. Statistical significance is indicated as **p < 0.01.

Given the compensatory up-regulation of *LvGon* observed under prolonged *LvIns* silencing, we next investigated whether *LvGon* is involved in the regulation of glucose metabolism in *L. vannamei*. *In vivo* experiments were conducted in which a high-concentration glucose solution was injected into the hemocoel of shrimp, and serum glucose levels were monitored over time. Serum glucose rapidly increased to >24 mmol/L within 5 min after injection and was subsequently cleared in a time-dependent manner, returning to basal physiological levels (~1 mmol/L) within 360 min (**Figure 4**, black line). Concomitant with the transient hyperglycemia, *LvGon* expression was significantly induced in all examined tissues, including the hepatopancreas, stomach, muscle, and hemocytes. In the hepatopancreas, *LvGon* transcript levels peaked at 15 min post-injection, reaching 3.65-fold of the basal level (**Figures 4A-a**). In contrast, peak induction of *LvGon* in the stomach, muscle, and hemocytes was observed a 30 min after glucose administration (**Figures 4A-b**,c,d).

To further substantiate the glucose responsiveness of *LvGon*, primary hepatopancreas cells of *L. vannamei* were cultured *in vitro* and exposed to exogenous glucose (**Figure 4B**). The results showed that intracellular glucose levels increased significantly following glucose treatment, consistent with the *in vivo* observations (**Figure 4B**, data within the white box). qRT-PCR analysis further revealed a rapid and significant induction of *LvGon* expression in response to glucose stimulation. Specifically, *LvGon* transcript levels increased to 2.42-, 1.83-, and 3.21-fold of control levels at 10, 20, and 40 min post-treatment, respectively (**Figure 4C**).

### 3.5 Effect of exogenous insulin administration on *LvGon* expression

**Figure 5:**
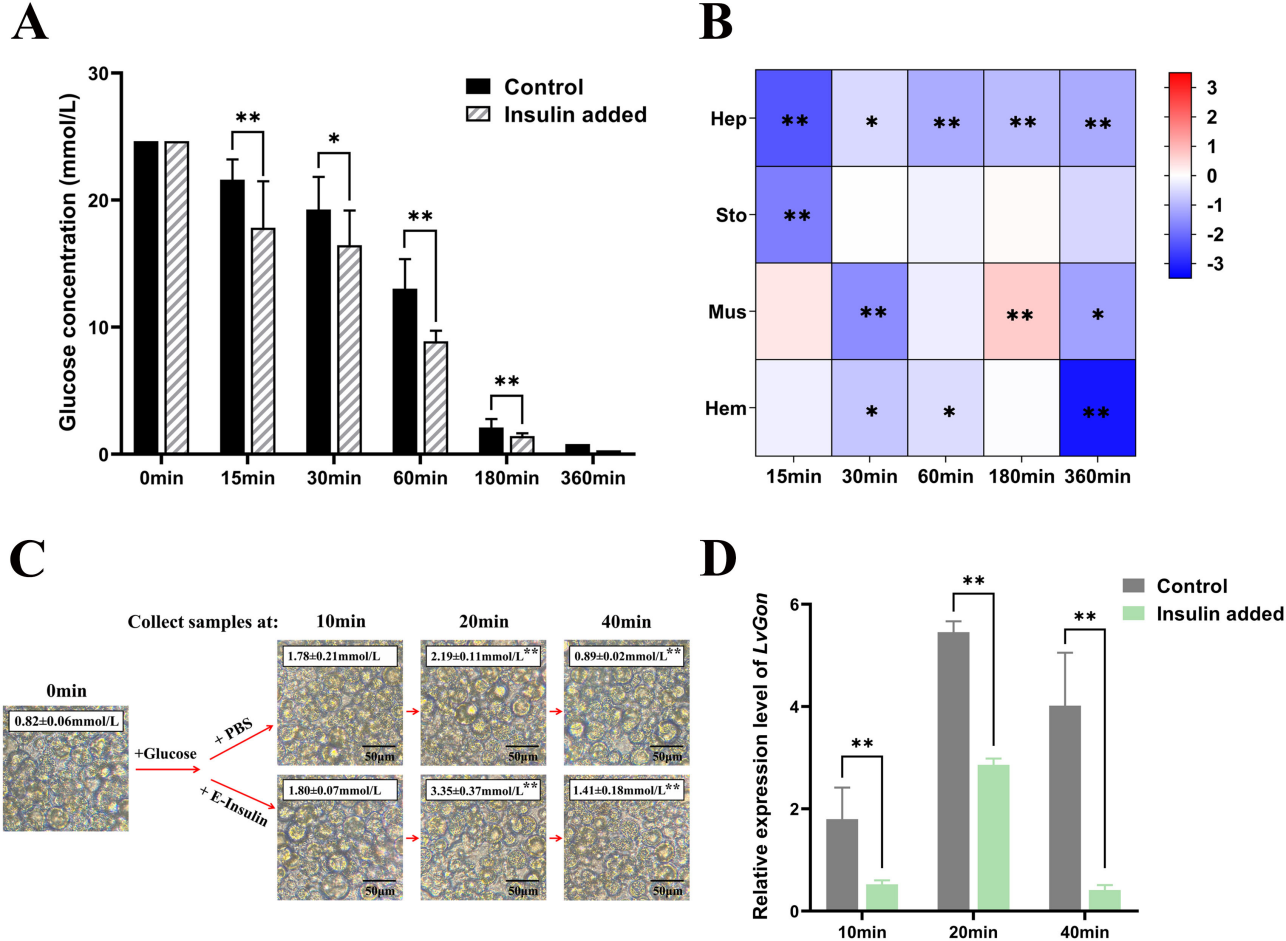
Effects of exogenous insulin administration on *LvGon* expression. **(A)** Changes in serum glucose concentration in the control and insulin-added groups following *in vivo* insulin injection. At each time point, data represent three biological replicates (12 shrimp per replicate). **(B)** Heatmap showing log_2_ fold changes (log_2_FC) in *LvGon* expression in hepatopancreas (Hep), stomach (Sto), muscle (Mus), and hemocytes (Hem) after insulin injection *in vivo*. Red and blue colors indicate up- and down-regulation, respectively. **(C)** Experimental design of the *in vitro* exogenous insulin treatment in primary hepatopancreatic cells and representative microscopic images. The values shown in the white boxes indicate the measured intracellular glucose concentrations. Asterisks (**) denote significant differences between insulin-added and control groups. **(D)** Relative expression levels of *LvGon* in primary hepatopancreatic cells following *in vitro* insulin treatment for different time points. For each treatment group at each time point, three independent replicate wells were established (derived from a mixed cell suspension of 12 shrimp per replicate; n = 3). Data are presented as mean ± SD. Statistical significance is indicated as *p < 0.05, **p < 0.01.

To further investigate the regulatory relationship between insulin signaling and *LvGon* expression, *in vivo* insulin administration experiments were conducted to examine the temporal expression dynamics of *LvGon* across multiple tissues. The results showed that exogenous insulin injection significantly accelerated glucose clearance in shrimp serum (**Figure 5A**). Within 360 min post-high glucose injection, the serum glucose level of the insulin injection group continued to be significantly lower than that of the control group, until there was no significant difference after 360 min (p > 0.05). Subsequent qRT−PCR analysis revealed significant suppression of *LvGon* expression in hepatopancreas, stomach, muscle and hemocytes (**Figure 5B**). Among these, the hepatopancreas exhibited the most pronounced and sustained inhibitory response, with *LvGon* transcript levels remaining significantly reduced from 15 to 360 min post-insulin injection, whereas stomach showed significant down-regulation primarily at 15 min, and hemocytes and muscle showed significant decreases at 30 and 360 min (**Figure 5B**).

To further validate the direct influence of insulin on *LvGon* expression, primary hepatopancreas cells were cultured *in vitro* and treated with exogenous insulin following glucose stimulation (**Figure 5C**). As expected, intracellular glucose levels in insulin-treated cells significantly exceeded those in control cells at 10, 20, and 40 min post-treatment (**Figure 5C**, data within the white box), confirming that exogenous insulin promoted cellular glucose uptake. This trend was consistent with the accelerated serum glucose clearance observed *in vivo*. Concurrently, insulin treatment significantly suppressed *LvGon* expression in hepatopancreas cells, with transcript levels reduced to 29.28%, 52.47%, and 29.47% of control values at 10, 20, and 40 min, respectively (**Figure 5D**).

### 3.6 Semi−quantitative tissue distribution of the four ILPs

**Figure 6:**
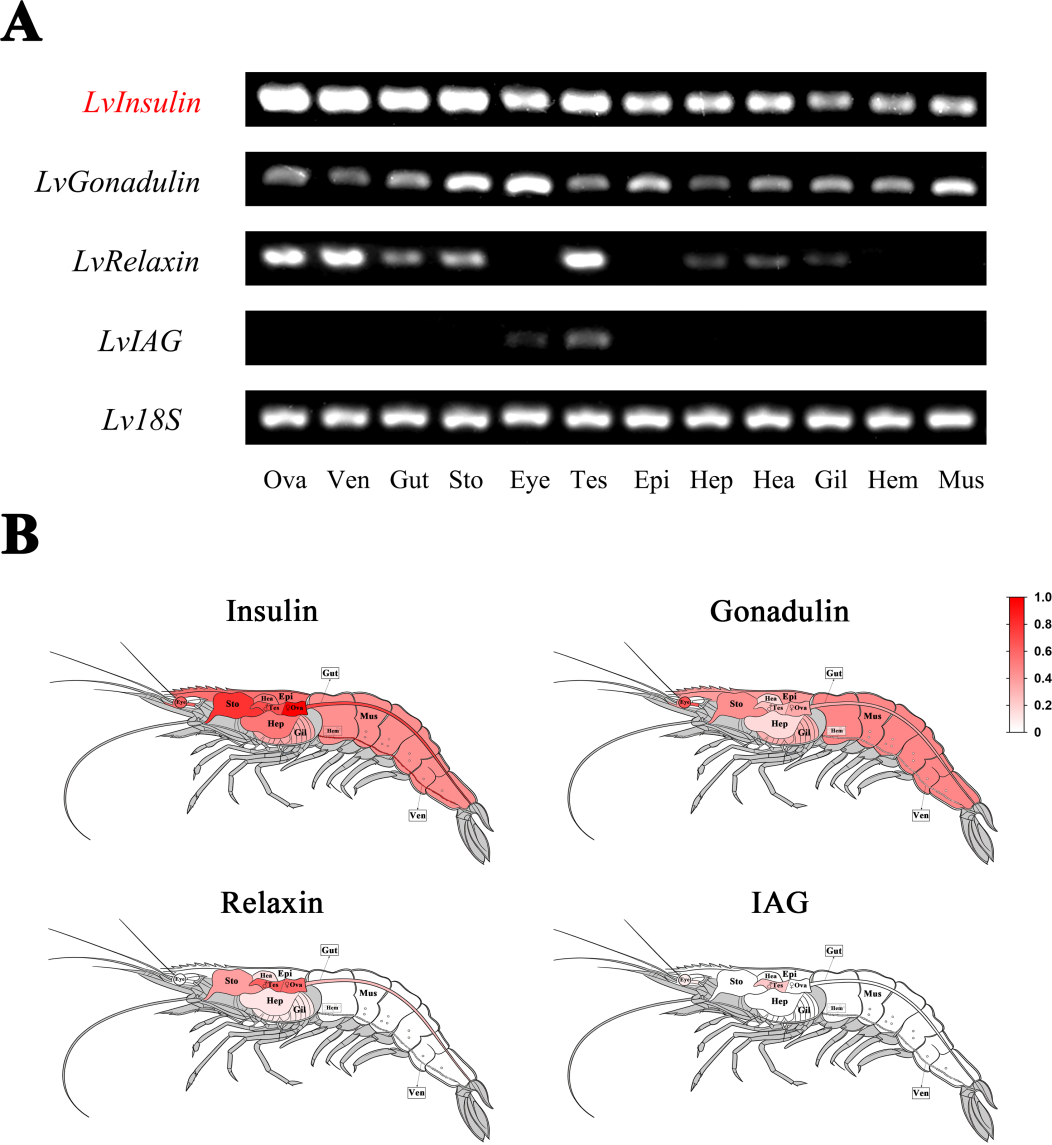
Tissue distribution patterns of four ILPs in *L. vannamei*. **(A)** Semi-quantitative PCR analysis showing the expression levels of four ILPs (*LvIns*, *LvGon*, *LvRelaxin*, and *LvIAG*) across 12 tissues of *L. vannamei*. *Lv18S* was used as the internal control. **(B)** Schematic representation of the tissue-specific expression distribution of the four ILPs in *L. vannamei.* Expression intensity is indicated by a color gradient ranging from white (no expression) to red (high expression), and brown regions denote tissues that were not examined. Tissue abbreviations: Ova, ovary; Ven, ventral nerve; Gut, gut; Sto, stomach; Eye, eyestalk; Tes, testis; Epi, epidermis; Hep, hepatopancreas; Hea, heart; Gil, gill; Hem, hemocytes; Mus, muscle.

In this study, semi-quantitative PCR was employed to profile the expression patterns of four ILP family members (*LvIns, LvGon, LvRelaxin* and *LvIAG*) across 12 tissues. The results revealed distinct tissue-specific expression patterns for each ILP (**Figure 6A**). Among them, *LvIns* exhibited the broadest distribution, with the highest overall expression across all tissues, with abundant expression in ovary, ventral nerve, gut and stomach. *LvGon* was another ILP that exhibited broad tissue distribution but at lower overall levels than *LvIns*, with particularly high expression in eyestalk, stomach, muscle and epidermis. In contrast, the expression of *LvRelaxin* and *LvIAG* exhibited significant tissue specificity. The expression of *LvRelaxin* was mainly concentrated in ovary, ventral nerve and testis and was undetectable in eyestalk, epidermis, hemocytes and muscle. Similarly, *LvIAG* expression was highly restricted to testis, with negligible presence in other tissues, and only weak expression detected in the eyestalk.

To further elucidate the differential expression patterns of these ILPs, we generated clustering heatmaps to visualize the tissue-specific expression of each ILP (**Figure 6B**). These heatmaps provided a clearer and more intuitive representation of the ILP expression patterns across tissues. The analysis revealed a striking similarity in the tissue expression patterns of *LvIns* and *LvGon*, suggesting potential functional overlap or coordinated regulation between these two ILPs.

### 3.7 Structural conservation of LvIns and LvGon and their receptor-binding potential

**Figure 7:**
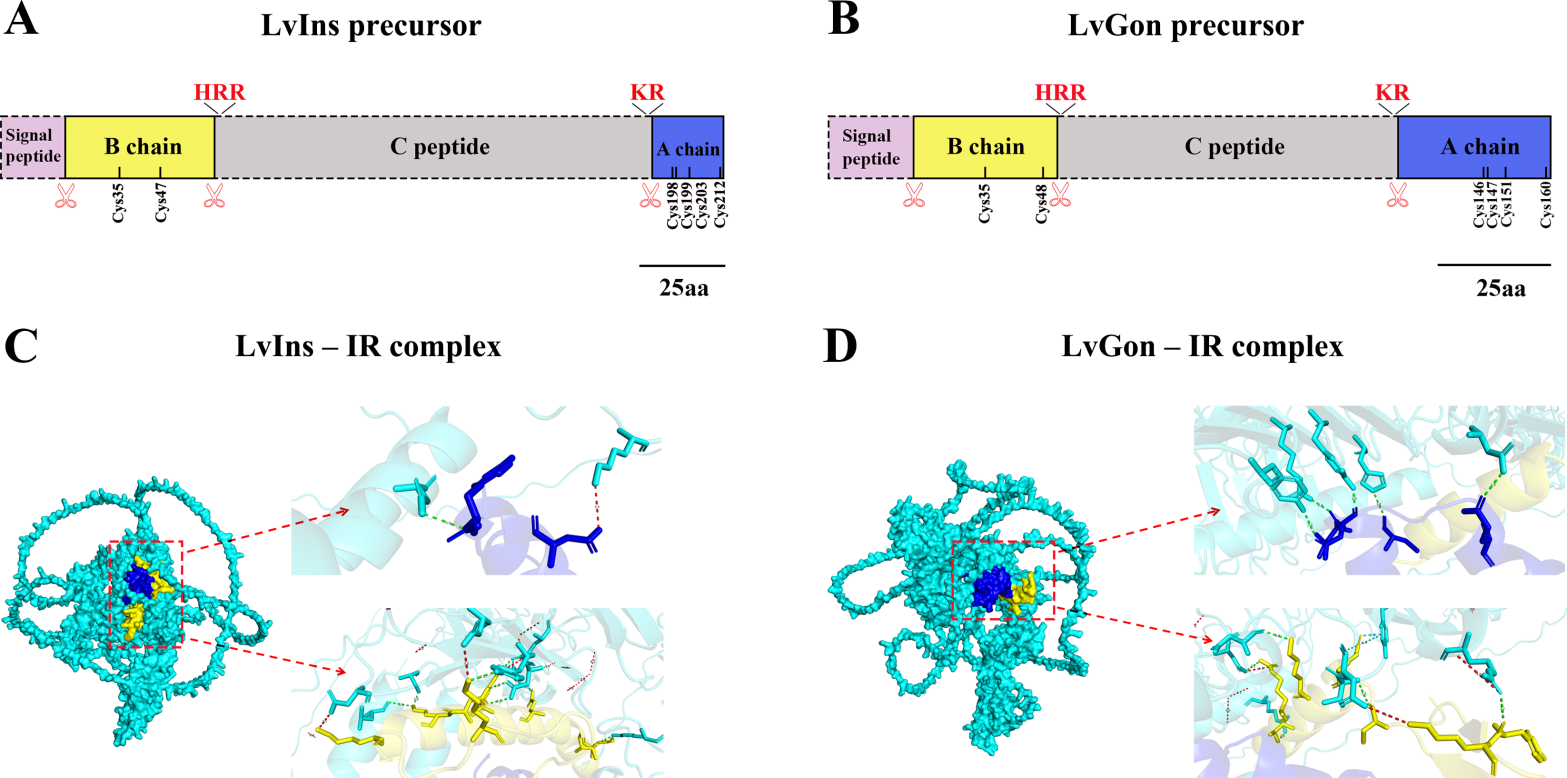
Structural features of LvIns and LvGon precursors and predicted interactions with the insulin receptor. **(A, B)** Schematic representation of the precursor protein structures of LvIns **(A)** and LvGon **(B)**. The signal peptide, B chain, C peptide, and A chain are indicated in pink, yellow, gray, and blue, respectively. Predicted pro-insulin cleavage sites (HRR and KR) are highlighted in red. **(C, D)** Predicted three-dimensional complexes of LvIns **(C)** and LvGon **(D)** with the insulin receptor (IR), showing the interaction interfaces between the A and B chains and the receptor. The IR is shown in cyan, while the A and B chains of LvIns/LvGon are colored blue and yellow, respectively. Key molecular interactions are indicated, including hydrogen bonds (green dashed lines), salt bridges (red dashed lines), and cation–π interactions (light blue dashed lines).

Protein sequence and structural analyses derived from nucleotide data indicate that both LvIns and LvGon exhibit typical structural characteristics of pro-insulin precursors. Specifically, both peptides contain an N-terminal signal peptide (19 amino acids for both), a B-chain (LvIns: 45 aa; LvGon: 31 aa), a C-peptide (LvIns: 128 aa; LvGon: 77 aa), and an A-chain (LvIns: 21 aa; LvGon: 34 aa). The presence of six conserved cysteine residues within the B- and A-chains in both peptides strongly suggests the formation of characteristic intra- and inter-chain disulfide bonds, a hallmark of functional insulin-like peptides. Notably, the same protease cleavage sites, “HRR” and “KR,” were identified in the connecting regions between the B-chain and C-peptide, as well as between the C-peptide and A-chain in both LvIns and LvGon (**Figures 7A,B**).

Molecular interaction simulations between LvIns and LvGon with the insulin receptor (IR) revealed that both peptides can specifically bind to the IR. For LvIns, a hydrogen bond (Cys21–1246) and a salt bridge (Cys18–1258) are formed between the A-chain of LvIns and the IR. The B-chain of LvIns forms a total of 10 hydrogen bonds (Cys34–961, Cys34–963, Cys29–1028, Cys22–974, Cys23–1233, Cys23–1237, Cys19–1036, Cys12–537, Cys4–1289, Cys7–1289) and three salt bridges (Cys19–1036, Cys29–1028, Cys34–961) (**Figure 7C**) with IR. Similarly, LvGon also forms multiple interactions with the IR. Specifically, five hydrogen bonds are formed between the A-chain of LvGon and the IR (Cys22–879, Cys21–854, Cys22–886, Cys19–888, Cys27–944). The B-chain of LvGon interacts with the receptor via five hydrogen bonds (Cys7–987, Cys16–1054, Cys22–949, Cys26–891, Cys30–1186), two salt bridges (Cys8–1055, Cys26–891), and one cation-π bond (Cys19–993) (**Figure 7D**).

### 3.8 Transcriptome analysis following *LvIns* RNA interference

To elucidate the molecular mechanisms underlying the reversal of growth rate in *L. vannamei* following prolonged *LvIns* silencing, transcriptome sequencing (RNA-Seq) was performed on the shrimp after 20 days of *LvIns* gene knockdown. A total of six sequencing libraries were generated, consisting of three biological replicates each from the dsLvIns interference and control groups, yielding 37.66 GB of clean data. Data quality assessment showed that the Q30 values ​​of all libraries exceeded 95.5% (**Table 2**), ensuring the reliability of the sequencing data for subsequent analysis. The raw sequencing data has been deposited in the NCBI SRA database (accession number: PRJNA1415973).

**Table 2:** An overview of sequencing and assembly of the transcriptome from *L. vannamei*.

Sample	Clean reads	Mapping rate(%)	GC(%)	≥ Q30(%)
Control-1	22422079	90.31	51.08	96.18
Control-2	20291791	89.73	50.75	95.80
Control-3	22356746	89.95	50.76	95.97
dsLvIns-1	20421096	89.44	50.32	95.72
dsLvIns-2	20550017	90.43	50.95	95.94
dsLvIns-3	19931391	90.09	50.28	95.56

**Figure 8:**
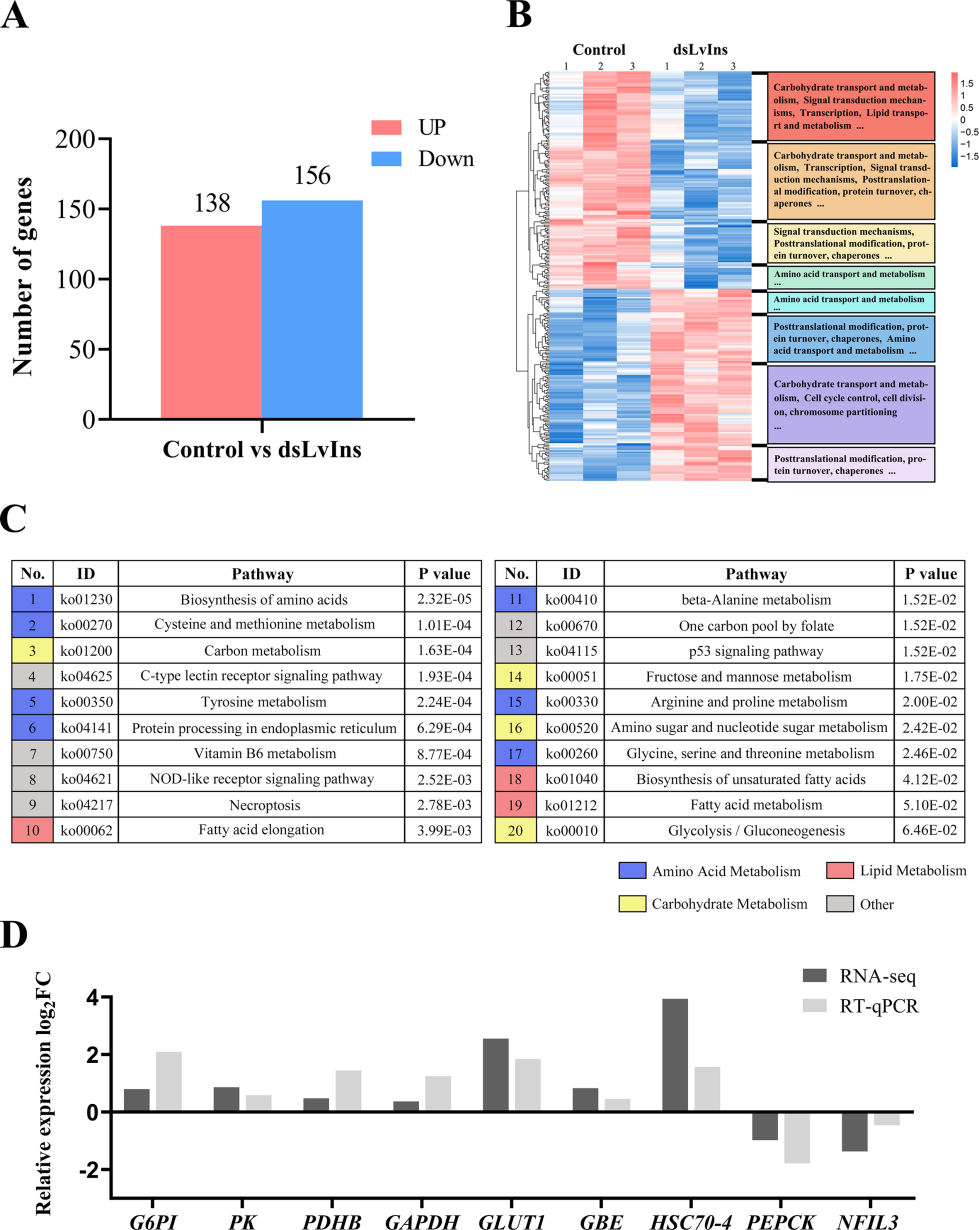
Transcriptomic analysis of the hepatopancreas after 20 days of *LvIns* interference. **(A)** Summary of differentially expressed genes (DEGs) identified between the control and dsLvIns groups. The numbers of up-regulated and down-regulated DEGs are indicated. **(B)** Heatmap showing the expression profiles of DEGs in the hepatopancreas. Rows represent genes and columns represent individual samples, with three biological replicates for both the control and dsLvIns groups. Gene expression levels are shown as log_10_-transformed FPKM values, with a color gradient ranging from blue (low expression) to red (high expression). Functional category enrichment of DEGs based on the eggNOG database is displayed on the right. **(C)** Top 20 significantly enriched KEGG pathways among the up-regulated DEGs. ID indicates the KEGG pathway identifier, Pathway denotes the pathway name, and P value represents the significance of enrichment. Pathways are color-coded according to their metabolic categories. **(D)** Validation of RNA-seq results by qRT-PCR. The log_2_ fold changes (log_2_FC) of nine selected genes measured by RNA-seq and qRT-PCR are compared. Positive and negative values indicate up-regulation and down-regulation, respectively.

Differential expression analysis, using thresholds of Fold Change ≥ 1.5 and False discovery rate (FDR) < 0.05, identified a total of 294 significantly differentially expressed genes (DEGs), with 138 genes up-regulated and 156 genes down-regulated (**Figure 8A**). Cluster heatmap analysis and EggNOG (Evolutionary Genealogy of Genes: Non-supervised Orthologous Groups) functional enrichment analysis revealed that these DEGs were significantly enriched in functional categories, including “carbohydrate transport and metabolism”, “amino acid transport and metabolism,” and “post-translational modifications, protein turnover, molecular chaperones” (**Figure 8B**). These findings suggest that *LvIns* silencing may modulate shrimp growth and metabolism by influencing genes involved in these critical biological processes. Furthermore, KEGG (Kyoto Encyclopedia of Genes and Genomes) pathway enrichment analysis highlighted that the most prominently up-regulated DEGs were associated with three major metabolic directions among the top 20 enriched pathways: 1) amino acid metabolism (e.g., biosynthesis of amino acids, cysteine and methionine metabolism pathways); 2) carbohydrate metabolism (e.g., carbon metabolism, glycolysis/gluconeogenesis pathway); 3) lipid metabolism (e.g., biosynthesis of unsaturated fatty acids, fatty acid metabolism pathways) (**Figure 8C**).

To validate the RNA-Seq results, qRT-PCR was performed to measure the expression levels of nine randomly selected genes. The qRT-PCR results were highly consistent with the RNA-Seq data, confirming the high accuracy and reliability of the transcriptomic analysis (**Figure 8D**).

### 3.9 Detailed expression profiles of key metabolic and growth signaling genes

**Figure 9:**
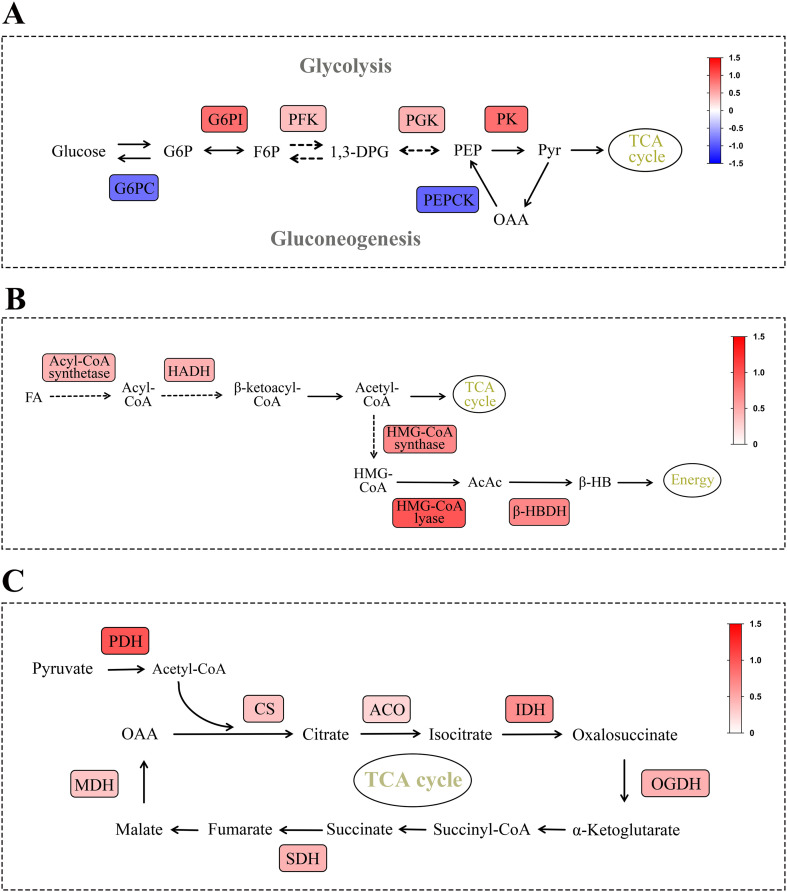
Transcriptional changes of key metabolic pathway–related genes in the hepatopancreas after 20 days of *LvIns* interference. **(A)** Schematic overview of the glycolysis and gluconeogenesis pathways. Nodes represent metabolites and arrows indicate enzymatic reaction steps. **(B)** Schematic overview of fatty acid metabolism and ketone body synthesis pathways. **(C)** Schematic overview of the tricarboxylic acid (TCA) cycle. For all panels, relative gene expression changes derived from the transcriptomic analysis are indicated by colored boxes corresponding to log_2_ fold changes (log_2_FC), with red representing up-regulated genes and blue representing down-regulated genes.

Based on the global transcriptome analysis, we further focused on key pathways related to energy homeostasis and growth regulation to characterize their transcriptional responses to long-term *LvIns* interference. The results confirm that prolonged interference with *LvIns* leads to a pronounced reprogramming of glucose metabolism in *L. vannamei*, consistent with the enzyme activity changes observed following prolonged interference. Specifically, genes encoding key enzymes of the glycolysis pathway were generally up-regulated, including glucose-6-phosphate isomerase (G6PI, LOC113819923, up-regulated 1.83-fold), phosphofructokinase (PFK, LOC113813801, 1.32-fold), phosphoglycerate kinase (PGK, LOC113825776, 1.39-fold), and pyruvate kinase (PK, LOC113828822, 1.82-fold). In contrast, the expression of rate-limiting enzymes involved in gluconeogenesis, such as glucose-6-phosphatase (G6PC, LOC113829848) and phosphoenolpyruvate carboxykinase (PEPCK, NewGene_933), was significantly down-regulated, with expression levels reduced to 55.20% and 53.36% of the control group, respectively (**Figure 9A**). Notably, these results are completely contrary to the gene expression profiles observed in the previous 12-day *LvIns* interference experiment (31).

In addition to glucose metabolism, long-term *LvIns* interference resulted in a substantial enhancement of fatty acid metabolic pathways (**Figure 9B**). Key enzymes involved in fatty acid β-oxidation, such as fatty acyl-CoA synthase (LOC113812015) and hydroxyacyl-CoA dehydrogenase (HADH, LOC113810046), exhibited expression increases of 1.36-fold and 1.39-fold, respectively, compared to the control group. Similarly, genes encoding enzymes in the ketone body production—such as HMG-CoA synthase (LOC113809364), HMG-CoA lyase (LOC113802152), and β-hydroxybutyrate dehydrogenase (β-HBDH, LOC113819961)—were significantly up-regulated, with expression levels increased by 1.64-fold, 2.10-fold, and 1.64-fold, respectively.

Consistent with these metabolic changes, the tricarboxylic acid (TCA) cycle, a central hub of cellular energy metabolism, exhibited coordinated up-regulation of multiple enzyme-coding genes following prolonged *LvIns* silencing (**Figure 9C**). Notably, increased expression was observed for citrate synthase (CS, LOC113815228; 1.29-fold), aconitate hydratase (ACO, LOC113810853; 1.22-fold), isocitrate dehydrogenase (IDH, LOC113807827; 1.59-fold), α-oxoglutarate dehydrogenase (OGDH, LOC113812483; 1.39-fold), succinate dehydrogenase (SDH, LOC113823464; 1.40-fold), and malate dehydrogenase (MDH, LOC113811537; 1.26-fold). Moreover, the expression of the pyruvate dehydrogenase E1 component subunit alpha (PDHA1, LOC113818169), which mediates the entry of pyruvate into the TCA cycle, was markedly increased by 2.05-fold.

To explore the molecular signaling mechanisms underlying the observed recovery of growth, we next examined key growth-regulatory pathways. Transcriptomic analysis revealed significant activation of the Wnt and MAPK/ERK signaling pathways (**Table 3**). Within the Wnt signaling pathway, genes encoding positive regulators, including Wnt ligands and Frizzled receptors, were up-regulated, whereas genes encoding negative regulators, such as glycogen synthase kinase-3β (GSK-3β) and adenomatous polyposis coli protein (APC), were down-regulated. Similarly, core components of the MAPK/ERK cascade, including EGFR, RAF, MEK, and ERK, exhibited a general trend toward increased expression. Collectively, these results indicate that compensatory activation of growth-related signaling pathways accompanies metabolic reprogramming and likely contributes to the recovery of growth in *L. vannamei* under prolonged *LvIns* deficiency.

**Table 3:** Expression changes of key regulatory factors in the Wnt and MAPK/ERK signaling pathways.

Pathway	Gene	ID	Control_Mean	dsLvIns_Mean	Fold Change	Regulated
Wnt	Wnt	LOC113816766	0.25	0.60	2.36	up
		LOC113827793	0.10	0.13	1.27	
		LOC113806005	2.07	2.53	1.22	
	LRP6	LOC113807131	0.57	0.78	1.37	up
	Frizzled	LOC113826281	0.52	0.63	1.21	up
	Dvl	LOC113828701	0.31	0.42	1.34	up
	BCL	LOC113827288	4.58	5.79	1.27	up
	Pygo	LOC113822876	0.49	0.97	1.97	up
	GSK3β	LOC113820486	9.90	9.08	0.92	down
	APC	LOC113804138	0.53	0.38	0.72	down
	Axin	LOC113802786	1.66	1.47	0.89	down
MAPK/ERK	EGFR	LOC113823280	0.03	0.09	3.66	up
	Raf	LOC113814953	1.11	1.33	1.20	up
	MEK1	NewGene_179	1.93	2.54	1.32	up
	ERK1/2	LOC113801198	1.83	2.08	1.14	up

Pathway indicates the signaling pathway name; Gene denotes the genes involved in the signaling pathways; ID refers to the corresponding NCBI accession number; Control_Mean and dsLvIns_Mean represent the FPKM values of the control and ds dsLvIns-treated groups in the transcriptome analysis, respectively; Fold Change indicates the expression change ratio between the experimental and control groups; Regulated denotes the gene is up-regulated or down-regulated.

## 4 Discussion

### 4.1 Endocrine resilience following sustained insulin-type ILP suppression in *L. vannamei*

Crustaceans represent a major group of economically important aquaculture species, and understanding the endocrine mechanisms underlying growth and metabolic regulation is of both fundamental and applied significance (39). Among these regulatory systems, ILP signaling has emerged as a central axis coordinating somatic growth, glucose metabolism, and reproductive development (4, 40). The identification of IAG as a key determinant of male sexual differentiation first underscored the importance of ILPs in crustaceans (41). Subsequent studies have revealed that multiple ILP subtypes collectively contribute to metabolic regulation and developmental coordination (22, 27).

Within the ILP family, insulin-type ILPs are of particular importance, as they are considered direct homologs of *Drosophila* DILP1–6 and are likely to occupy central positions in systemic growth and metabolic regulation (31, 42). In *Drosophila*, growth control is predominantly mediated by DILP1-6, with DILP2 functioning as the principal circulating growth factor (43). Conversely, DILP7 and DILP8—homologous to relaxin-type and Gon/IAG-type ILPs in crustaceans—are more closely linked to reproductive coordination and developmental timing (44, 45). This functional hierarchy implies that disruption of insulin-type ILPs would be expected to result in sustained growth impairment.

Consistent with this expectation, both our previous and current experiments demonstrated that short-term (12 days) *LvIns* knockdown results in significant growth suppression and reduced hepatopancreatic glucose levels (31). However, a marked physiological transition emerged under prolonged interference (20 days). Despite sustained *LvIns* suppression, growth inhibition did not progressively intensify. Instead, growth performance and metabolic parameters gradually recovered, with growth rate in the interference group ultimately exceeding that of both the PBS and dsEGFP controls during the late phase of treatment. Concurrently, hepatopancreatic glucose levels rebounded and glycolytic enzyme activities increased, whereas gluconeogenic activity declined, indicating a coordinated metabolic reprogramming.

Importantly, *LvIns* expression remained consistently suppressed throughout the experimental period, excluding RNAi inefficiency as an explanation for this recovery. Rather, the transition from early growth inhibition to subsequent recovery indicates activation of intrinsic compensatory mechanisms. This dynamic adaptation reflects a form of endocrine resilience, whereby the organism buffers metabolic instability under sustained disruption of a key regulatory ligand and restores physiological homeostasis.

Compensatory regulation of insulin signaling is a conserved phenomenon across metazoans. In mammals, chronic insulin resistance triggers β-cell expansion and enhanced insulin secretion to preserve glucose homeostasis (46). In invertebrates possessing expanded ILP families, compensation more commonly occurs through transcriptional up-regulation of paralogous ligands (42). In *Drosophila*, loss of specific DILPs induces expression of alternative family members to maintain pathway integrity (16). Similar compensatory responses have been documented in other insects, including up-regulation of *CmILP3* following *CmILP1* or *CmILP2* silencing in *C. maculatus* (17), and increased *BgILP3* expression after *BgILP5* knockdown in *B. germanica* (18). Although the specific ligands involved differ among taxa, a common principle emerges: preservation of insulin signaling integrity is essential for maintaining systemic homeostasis.

Our findings extend this principle to crustaceans. The recovery of growth and metabolic parameters following prolonged *LvIns* suppression suggests that gene family expansion confers functional robustness to the crustacean ILP network. A similar phenomenon has been reported in *M. rosenbergii*, where continuous ILP knockdown for 21 days reduced *MrILP* expression, but paradoxically increased hemolymph glucose levels in the interference group and enhanced the activities of key enzymes involved in both glycolysis and gluconeogenesis (25). Although the underlying mechanisms were not investigated in that study, the convergence of these observations across decapod species points to a conserved adaptive strategy within crustaceans.

Notably, sustained *LvIns* interference induced a marked up-regulation of *LvGon* (4.30-fold), suggesting coordinated transcriptional rebalancing within the ILP network. Based on these observations, we hypothesize that prolonged interference of insulin-type ILP may activate a compensatory endocrine module, potentially involving the up-regulation of Gon-type ILP, which could restore IIS activity and stabilize metabolic homeostasis. This working hypothesis provides a conceptual framework for further dissecting network-level redundancy and regulatory plasticity within the crustacean ILP system.

### 4.2 Regulatory role of Gon-type ILP (*LvGon*) in glucose homeostasis in *L. vannamei*

To further evaluate the compensatory hypothesis proposed above, we investigated whether *LvGon* participates in glucose metabolic regulation in *L. vannamei*. Although Gon-type ILPs were originally named following their identification in the gonads of *P. japonicus*, their broader physiological functions in crustaceans remain incompletely defined (26). Given the pronounced up-regulation of *LvGon* under prolonged *LvIns* suppression, we hypothesized that *LvGon* may contribute to systemic glucose homeostasis. To test this, we combined glucose challenge assays and exogenous insulin administration with *in vivo* and *in vitro* analyses to characterize the expression dynamics and functional responsiveness of *LvGon*.

Following intrahaemocoelic injection of a high-concentration glucose solution, serum glucose levels increased sharply within 5 min, and gradually returned to baseline within 360 min, indicating an efficient glycemic regulatory capacity in shrimp. Concomitantly, *LvGon* expression was significantly up-regulated across multiple metabolically active tissues, including the hepatopancreas, stomach, muscle, and hemocytes. Consistent with the *in vivo* observations, primary hepatopancreatic cell cultures exposed to high glucose also exhibited rapid and significant induction of *LvGon* expression. The rapid onset and tissue-wide induction of *LvGon* suggest that it functions as a glucose-responsive endocrine factor rather than a gonad-restricted peptide. This interpretation is also supported by previous studies showing that Gon-type ILPs are involved in glucose metabolic regulation in *P. trituberculatus* and *M. rosenbergii*, indicating that this peptide class may have broader physiological functions beyond reproduction (25, 27).

To further clarify its position within the insulin signaling network, we evaluated *LvGon* expression following exogenous insulin administration. Insulin treatment significantly accelerated glucose clearance, as reflected by persistently lower serum glucose levels in treated shrimp compared with controls over a 6-hour period. Notably, *LvGon* expression was significantly suppressed across all examined tissues. This inhibitory effect was further confirmed *in vitro*, where exposure of primary hepatopancreatic cells to insulin resulted in a marked reduction in *LvGon* expression. These reciprocal regulatory patterns indicate that *LvGon* is negatively regulated by exogenous insulin and is responsive to systemic glycemic status. Such bidirectional responsiveness is consistent with the canonical feedback organization of insulin signaling pathways (27). Together, these dynamics suggest that *LvGon* may function within a glucose–insulin feedback loop in crustaceans.

Importantly, the dynamic expression profile of *LvGon* closely parallels that previously reported for *LvIns* under identical experimental conditions (31). Both peptides are induced by hyperglycemic stimulation and suppressed following exogenous insulin administration, revealing highly concordant regulatory patterns. This convergence in responsiveness provides mechanistic support for the compensatory hypothesis proposed above, whereby *LvGon* and *LvIns* may function in a coupled or partially redundant manner to preserve glucose homeostasis under sustained endocrine perturbation.

### 4.3 Compensatory mechanism of ILPs in *L. vannamei* under gene duplication

In this study, a series of experiments demonstrated that Gon-type ILPs participate in the regulation of glucose metabolism in *L. vannamei*. Notably, the expression pattern of *LvGon* in glucose homeostasis regulation closely resembles that of *LvIns* (31). Together with existing evidence, this finding suggests substantial functional overlap among crustacean ILP subtypes in regulating hemolymph glucose, a core physiological function. In *L. vannamei*, injection of recombinant *LvRelaxin* protein rapidly reduces hemolymph glucose levels, while knockdown of *LvRelaxin* inhibits shrimp growth and molting rates (28, 47). Similarly, in *Callinectes sapidus* and *Eriocheir sinensis*, knockdown of *IAG* or injection of recombinant *IAG* protein causes significant alterations in hemolymph glucose levels (35, 48). These converging findings suggest that functional redundancy among different ILP subtypes in crustaceans may cooperate to regulate glucose metabolism, reflecting the evolutionary conservation of insulin signaling during gene family expansion.

Gene family expansion is a key evolutionary mechanism that drives functional redundancy, enhancing the stability of physiological processes through gene dosage effects (49). Gene-level compensatory mechanisms are widely observed across both plants and animals. For example, in zebrafish, mutations in the endothelial extracellular matrix gene *EGFL7* did not result in a significant phenotype alterations, as the expression of its functionally similar gene *Emilin* was up-regulated, compensating for the loss of *EGFL7* function (50). Similarly, in *Pleurodeles waltl*, knockout of the regeneration-related gene *YAP* did not affect limb regeneration, because its homolog *TAZ* was specifically up-regulated, compensating for the loss of *YAP* (51). These examples demonstrate that functional redundancy, driven by gene duplication and homologous gene networks, provides effective buffering and compensatory mechanisms for organisms to cope with gene function loss.

The ILP family in decapod crustaceans has undergone typical gene duplication, resulting in the four known subtypes (21). In the present study, semi-quantitative tissue expression profiling revealed distinct but partially overlapping expression patterns among these subtypes. Specifically, *LvIns* exhibited a typical broad expression pattern, with high and widespread expression across the 12 tissues examined, suggesting its central role in maintaining basal physiological functions. Gon-type ILP showed a similar broad expression pattern, albeit at relatively lower levels, suggesting potential functional complementarity between the two peptides. In contrast, relaxin-type and IAG-type ILPs displayed more tissue-specific expression, with a primary focus on gonadal tissues (testes and ovaries), consistent with their specialized roles in sex differentiation and reproductive regulation (41). The broad expression profiles of insulin-type and Gon-type ILPs suggest that their functions extend beyond sex-regulation, potentially playing fundamental roles in maintaining systemic metabolic homeostasis.

Protein structure analysis further supports the potential compensation between *LvIns* and *LvGon*. Both peptides exhibit a typical pre-proinsulin structure, including structurally similar A-chain, B-chain, C-peptide, proteolytic cleavage sites, and six conserved cysteine residues. These features indicate that both peptides may undergo similar post-translational processing, including signal peptide cleavage, C-peptide removal, and disulfide bond formation, to form functional mature peptides (7). Furthermore, molecular docking simulations revealed that the mature peptides of both ILPs can bind to the insulin receptor through similar spatial conformations, forming stable interactions via hydrogen bonds, salt bridges, and other non-covalent forces. Sequence and structural similarity represent important prerequisites for potential functional compensation between paralogous genes (52). This implies that, under conditions of prolonged interference of insulin-type ILPs, up-regulation of Gon-type ILPs could occupy the same receptor-binding sites and activate conserved downstream signaling pathways, thus achieving functional compensation.

In summary, our study systematically analyzes the functional roles, tissue expression patterns, protein structures, and receptor-binding potential between *LvIns* and *LvGon*. The findings suggest that *LvIns* and *LvGon* share high similarity in all of these characteristics, supporting the potential for functional compensation between the two peptides within the crustacean ILP system.

### 4.4 Transcriptomic insights into the molecular basis of insulin signaling compensation following prolonged *LvIns* suppression

To further elucidate the molecular mechanisms underlying the long-term compensatory response to prolonged *LvIns* interference, we performed transcriptomic profiling of hepatopancreas tissue after 20 days of RNAi, using PBS-injected shrimp as controls. The hepatopancreas, a central organ in crustaceans, regulates digestion and metabolism, allocating nutrients and energy to peripheral tissues, such as muscle and gonads, during growth and reproduction (53). Comparative transcriptome analysis identified 294 differentially expressed genes (DEGs) between the interference and control groups. Functional categorization revealed that the DEGs were predominantly enriched in pathways associated with “carbohydrate transport and metabolism”, “amino acid transport and metabolism”, and “post-translational modification and protein turnover”. KEGG pathway enrichment further indicated that carbohydrate, amino acid, and lipid metabolic pathways constituted the majority of significantly up-regulated pathways, consistent with the expected global metabolic remodeling induced by sustained *LvIns* suppression.

A major feature of this transcriptomic response was the reversal of the metabolic imbalance observed during short-term *LvIns* interference. In our previous 12-day experiment, *LvIns* knockdown was associated with suppression of glycolytic genes and activation of gluconeogenic pathways. However, after 20 days of interference, a clear compensatory shift was observed. Key glycolytic rate-limiting enzymes—including G6PI, PFK, PGK, and PK—were significantly up-regulated, while the principal gluconeogenic genes G6PC and PEPCK were markedly down-regulated. This transcriptional rebalancing is consistent with the restored hepatopancreatic glucose levels observed phenotypically and suggests that compensatory endocrine signaling, potentially mediated by up-regulation of Gon-type ILP, reprograms carbohydrate metabolism by enhancing glycolytic flux and ATP production. This mechanism likely alleviates the chronic energy deficit caused by sustained suppression of insulin signaling.

In addition to carbohydrate metabolism, lipid metabolic pathways were prominently activated under prolonged *LvIns* interference. Genes encoding acyl-CoA synthetase and HADH, key enzymes in fatty acid activation and β-oxidation, were significantly up-regulated, indicating enhanced lipid catabolism. Concurrently, enzymes involved in ketone body synthesis and utilization—including HMG-CoA synthase, HMG-CoA lyase, and β-HBDH—were also up-regulated. These changes suggest that the hepatopancreas may compensate for sustained insulin-like signaling perturbation by mobilizing lipid-derived substrates and providing alternative energy sources. Consistent with this interpretation, multiple TCA cycle genes, including *CS*, *IDH*, *OGDH*, and *MDH*, were up-regulated, indicating reinforcement of central energy metabolism. The coordinated activation of glycolysis, fatty acid oxidation, ketogenesis, and the TCA cycle supports a systemic metabolic reprogramming process aimed at restoring bioenergetic capacity.

Transcriptomic data also suggested activation of growth-promoting signaling pathways under compensatory conditions. In the Wnt pathway, positive regulators, including Wnt ligands, Frizzled receptors, and *Dishevelled* were up-regulated, whereas inhibitory components such as *GSK-3β*, *APC*, and *Axin* were down-regulated. Similarly, the MAPK/ERK cascade showed an overall activation trend, with increased expression of core components such as *EGFR*, *RAF*, *MEK1*, and *ERK1/2*. Notably, *EGFR* expression was markedly elevated (3.66-fold). Given the established roles of Wnt and MAPK/ERK signaling in cell growth, proliferation, and tissue development (54–57), their activation may contribute to the observed growth rebound following prolonged *LvIns* suppression.

Taken together, these findings reveal a coordinated transcriptional reprogramming process that transitions the organism from metabolic compensation to growth restoration during prolonged insulin signaling suppression. On one hand, coordinated activation of glycolysis, lipid oxidation, ketogenesis, and the TCA cycle resolves the chronic energy deficit caused by *LvIns* interference. On the other hand, activation of growth-promoting pathways, including Wnt and MAPK/ERK, reinitiate growth programs once energetic stability is restored. These results highlight a multilayered compensatory mechanism in which endocrine adaptation, metabolic reprogramming, and growth signaling converge to restore physiological homeostasis under sustained perturbation of insulin-like signaling.

## 5 Conclusion

This study was initiated by an unexpected experimental observation and subsequently evolved into a systematic investigation of the compensatory dynamics within the crustacean ILP system under prolonged depletion of insulin-type ILP. Our findings collectively delineate a multilayered adaptive response that restores physiological homeostasis following sustained endocrine perturbation. Despite sustained *LvIns* knockdown, shrimp exhibited recovery of growth and glucose metabolism, accompanied by marked up-regulation of the Gon-type ILP *LvGon*. The similar glucose/insulin responsiveness, broad tissue distribution, conserved insulin-like structure, and predicted receptor-binding potential of *LvIns* and *LvGon* suggest that *LvGon* may partially compensate for reduced *LvIns* signaling.

**Figure 10:**
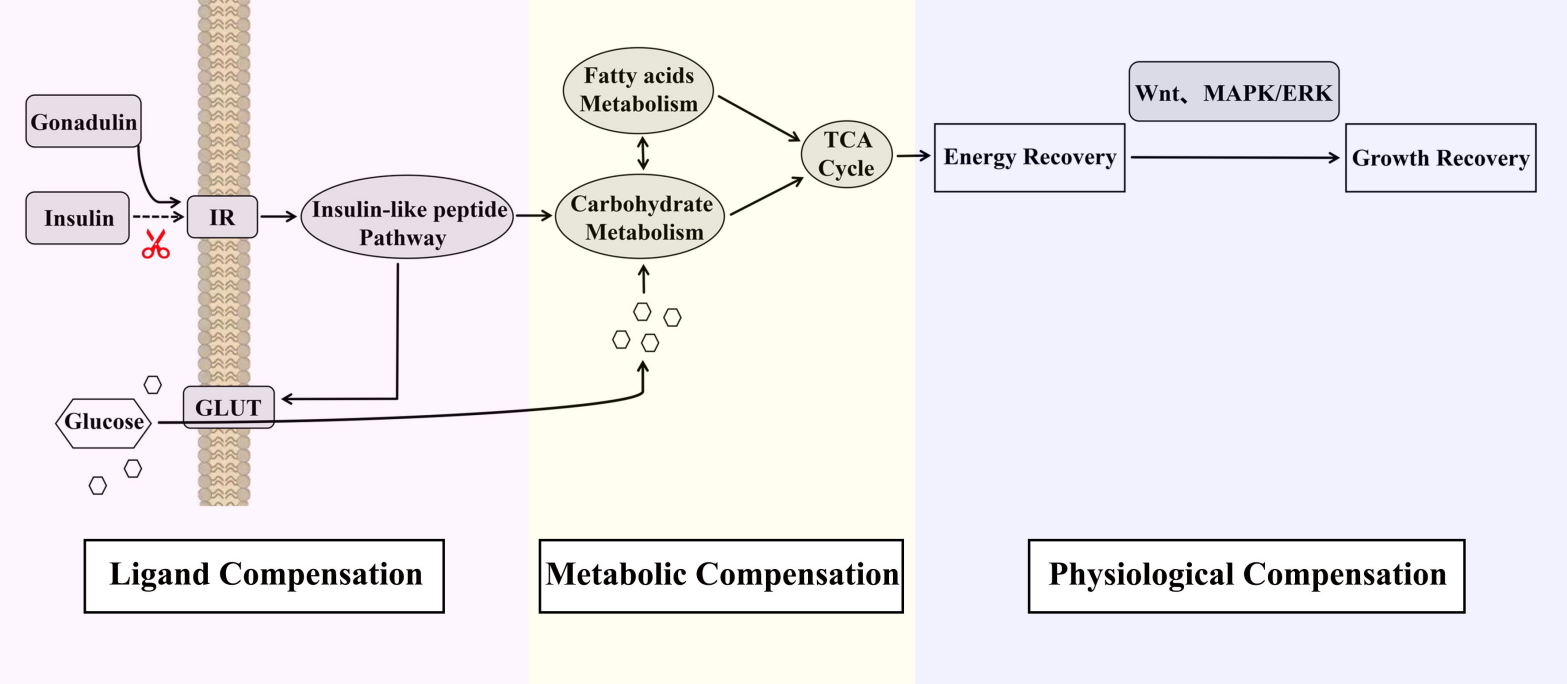
Proposed multilevel compensatory model between insulin-type and Gon-type ILPs in decapod crustaceans. The schematic illustrates a hierarchical compensatory framework activated under sustained depletion of insulin-type ILP. Distinct background colors delineate three interconnected layers of compensation: ligand compensation, metabolic compensation, and physiological compensation.

In parallel, prolonged *LvIns* interference induced coordinated metabolic reprogramming in the hepatopancreas, including activation of glycolysis, lipid catabolism, ketogenesis, and the TCA cycle, together with up-regulation of growth-associated Wnt and MAPK/ERK pathways. Collectively, these ligand-level, metabolic, and physiological adaptations constitute an integrated resilience strategy that enables crustaceans to buffer prolonged disruption of a central growth-regulatory factor. This proposed compensatory framework between insulin-type and Gon-type ILPs in decapod crustaceans is summarized in **Figure 10**. To our knowledge, this study provides the first evidence of functional compensation among ILP family members in crustaceans and offers new insight into endocrine redundancy and network plasticity in invertebrates.

## Data Availability

The datasets presented in this study can be found in online repositories. The names of the repository/repositories and accession number(s) can be found in the article/supplementary material.
